# Editorial: Autism Spectrum Disorders and Metal Dyshomeostasis

**DOI:** 10.3389/fnmol.2022.861483

**Published:** 2022-02-18

**Authors:** Mukesh K. Pandey, Andreas M. Grabrucker, Sunil Q. Mehta, Robert J. Harvey

**Affiliations:** ^1^Department of Radiology, Mayo Clinic, Rochester, MN, United States; ^2^Department of Biological Sciences, University of Limerick, Limerick, Ireland; ^3^Health Research Institute, University of Limerick, Limerick, Ireland; ^4^Bernal Institute, University of Limerick, Limerick, Ireland; ^5^Department of Pediatric and Adolescent Medicine, Mayo Clinic, Rochester, MN, United States; ^6^Department of Psychiatry and Psychology, Mayo Clinic, Rochester, MN, United States; ^7^School of Health and Behavioural Sciences, University of the Sunshine Coast, Maroochydore, QLD, Australia

**Keywords:** autism spectrum disorders, biometals, zinc, copper, selenium, serum analysis

Autism spectrum disorder (ASD) is a multifaceted neurodevelopmental disorder with a complex etiology that appears to be a mixture of underlying environmental and genetic risk factors. Among the environmental risk factors is perinatal metal dyshomeostasis. It is now evident that deficiency or dyshomeostasis of essential metal ions such as iron, zinc, and copper (Fe, Zn, and Cu) will affect the neurodevelopmental process that may not be corrected even after the repletion of these metal ions. In addition, other essential trace metals, such as manganese and molybdenum (Mn and Mo) and trace elements such as selenium (Se) ions, also play a critical role in neurodevelopment. Despite growing research on this subject, the key molecular and cellular mechanisms and the identification of metallome biomarkers remain to be elucidated. Consequently, research addressing the impact of Zn, Cu, Se, and Fe deficiencies in the ASD population will help to unravel ASD pathophysiology, deepen our knowledge of environmental co-factors, and serve as means of developing novel treatments or disease-modifying strategies. This is a rapidly emerging field of neuroscience research that is highlighted in the current Research Topic that is dedicated to understanding the impact of metal dyshomeostasis in ASD.

The role of the metallome and its relationship to other “omes” of the body is explored by Stanton et al., who explain the role of the metallome in ASD and how this is functionally and pathophysiologically linked to the transcriptome, proteome, microbiome, metabolome, and lipidome. This review highlighted the need to simultaneously integrate data exploring multiple “omes” to find ASD-specific signatures and possibly biomarkers for ASD. Moreover, Stanton et al. proposed a new model that may explain independently reported pathologies in ASD.

Consistent with the importance of the metallome in ASD, Behl et al. summarized the prevalence of abnormal levels of various metal micronutrients in ASD using hair, nail, and serum samples. In this perspective, Behl et al. described the critical role of Fe, Mg, Zn, Cu, and Se in ASD and expressed concerns over data variability by geographical locations. The authors suggested a critical need to stratify ASD-related biometal data by sex and age to draw more meaningful conclusions.

Mehta et al. reported on a study involving 64 healthy control and 65 ASD children in the 2–4-year-old age group. Using inductively coupled plasma mass spectrometry, the authors analyzed hair, nail, and serum matrixes to quantify Zn, Cu, and Se biometals. The team at the Mayo Clinic reported a significantly lower level of serum Se (116.83 ± 14.84 ng/mL) in male ASD cases compared to healthy male controls (128.21 ± 9.11 ng/mL; *p* < 0.005). The authors have also noticed a similar trend of lowered serum Se in nails of ASD children compared to controls. The finding of lower levels of serum Se in young ASD children (2–4 years) is very informative, as it is suggestive of a critical role for Se in early brain development.

Among the other articles included in this issue, Xia et al. studied the altered relationship between parvalbumin+ neurons (PV) and perineuronal nets (PNN) in a valproic acid (VPA)-based mouse model of ASD. The authors reported a decrease in PV intensity but an increase in PNN intensity in the VPA mouse model of ASD. They also established a relationship between PV and PNN intensities, showing that reducing PNN levels using *in vivo* injection of chondroitinase ABC corrected PV expression in adult VPA mice. Based on their findings, the authors concluded that a disruption between PV and PNN interactions resulted in ASD pathology.

By contrast, Shiva et al. focused on the expression of ermin (*ERMN*) and listerin E3 ubiquitin protein ligase 1 (*LTN1*) genes in the Iranian ASD population. These proteins are involved in oligodendrocyte maturation and protein degradation, and the cognate genes show altered expression in response to neuroinflammation. The authors reported a significant down-regulation of *ERMN* in the ASD group compared to healthy controls (posterior beta = −0.794, adjusted *P* = 0.025) and highlighted that gender-based data analysis showed significant down-regulation of *ERMN* expression in the male ASD group only (*r* = −0.49, *P* < 0.0001). This data provides further support for a critical role for ERMN and LTN1 in the pathogenesis of ASD in males. Tamizkar et al. studied the dysregulation of nuclear factor NF-kB-associated lncRNAs in ASD, examining the hypothesis that NF-κB affects the neuroinflammatory responses in the orbitofrontal cortex of ASD patients. The authors measured the expression of NF-κB-associated long non-coding RNAs (lncRNAs) and mRNAs in the peripheral blood of ASD and control groups, revealing increased expression of *ADINR, ANRIL, DILC, NKILA*, and *CHAST* in the ASD group compared with healthy controls. This suggests that NF-κB-associated lncRNAs might be playing a role in ASD pathogenesis. These two articles provide evidence that transcriptional responses to neuroinflammation are observable in patients with ASD.

In summary, this Research Topic brings together evidence for the role of various biometals (Zn, Fe, Cu) and lowered levels of serum Se in ASD. Collectively, these research articles also highlight the role of neuroinflammation, parvalbumin (PV), and perineuronal nets (PNN) in ASD, in addition to genetic factors. The future of research on the interaction of genetic and non-genetic factors in ASD holds promise for identifying novel therapeutic and preventive approaches. Further research aiming to understand, measure, and modify metal homeostasis in the body and brain has enormous potential for identifying novel ASD biomarkers and treatments.

## Author Contributions

All authors listed have made a substantial, direct, and intellectual contribution to the work and approved it for publication.

## Conflict of Interest

The authors declare that the research was conducted in the absence of any commercial or financial relationships that could be construed as a potential conflict of interest.

## Publisher's Note

All claims expressed in this article are solely those of the authors and do not necessarily represent those of their affiliated organizations, or those of the publisher, the editors and the reviewers. Any product that may be evaluated in this article, or claim that may be made by its manufacturer, is not guaranteed or endorsed by the publisher.

